# Presence of *Mycobacterium avium* subsp. *paratuberculosis* (MAP) in Brazilian patients with inflammatory bowel diseases and in controls: Presença de *Mycobacterium avium* subsp. *paratuberculosis* (MAP) em pacientes brasileiros com doença inflamatória intestinal e em controles

**DOI:** 10.1590/1516-3180.2014.8251809

**Published:** 2015-07-03

**Authors:** Isabel Azevedo Carvalho, David Germano Gonçalves Schwarz, Pricila Aparecida Grasse Pietralonga, Ana Carolina Silva Faria, Isis Freitas Espechit Braga, Gabriel Domingos Carvalho, Fabrício Luciani Valente, João Paulo Machado, Lize Maciel Pinheiro Guimarães, Maria de Lourdes Abreu Ferrari, Abelardo Silva, Maria Aparecida Scatamburlo Moreira

**Affiliations:** I DVM, PhD. Collaborator, Veterinary Department, Universidade Federal de Viçosa (UFV), Minas Gerais, Brazil.; II DVM. Doctoral Student, Veterinary Department, Universidade Federal de Viçosa (UFV), Viçosa, Minas Gerais, Brazil.; III DVM, MSc. Collaborator, Veterinary Department, Universidade Federal de Viçosa (UFV), Viçosa, Minas Gerais, Brazil.; IV DVM. Master’s Student, Veterinary Department, Universidade Federal de Viçosa (UFV), Viçosa, Minas Gerais, Brazil.; V DVM, PhD. Professor, Veterinary Department, Instituto Federal do Norte de Minas (IFNMG), Salinas, Minas Gerais, Brazil.; VI DVM, PhD. Postdoctoral Student, Veterinary Department, Universidade Federal de Viçosa (UFV), Viçosa, Minas Gerais, Brazil.; VII MD. Resident Physician, Medical School, Universidade Federal de Minas Gerais (UFMG), Belo Horizonte, Minas Gerais, Brazil.; VIII MD, PhD. Professor, Medical School, Universidade Federal de Minas Gerais (UFMG), Belo Horizonte, Minas Gerais, Brazil.; IX DVM, PhD. Professor, Veterinary Department, Universidade Federal de Viçosa (UFV), Viçosa, Minas Gerais, Brazil.

**Keywords:** Mycobacterium avium subsp, paratuberculosis, Crohn disease, Inflammatory bowel diseases, Colitis, ulcerative, Brazil

## Abstract

**CONTEXT AND OBJECTIVE:**

*Mycobacterium avium* subsp. *paratuberculosis* (MAP) has attracted the interest of researchers because of similarities between paratuberculosis and Crohn’s disease (CD). The aim of this study was to evaluate the frequency of MAP through cultures, histology and polymerase chain reaction (PCR) on intestinal biopsies from Brazilian CD patients. Quantitative real time PCR (qRT-PCR) was performed on positive samples.

**DESIGN AND SETTING:**

Analytical cross-sectional study with control group at two federal universities.

**METHODS:**

Fresh samples were collected from 25 patients; five with CD, eight with ulcerative colitis (UC) and 12 controls with non-inflammatory bowel disease (nIBD). Formalin-fixed paraffin-embedded (FFPE) samples from 143 patients were also collected: 44 CD, 49 UC and 56 nIBD.

**RESULTS:**

None of the fresh samples was positive for MAP. Five FFPE samples (one CD, two UC and two nIBD) and three fresh samples (one in each group) were positive through IS*900*-PCR. qRT-PCR was performed on these eight samples. Among the FFPE samples, there were 192.12 copies/µl in the CD group, 72.28 copies/µl in UC and 81.43 copies/µl in nIBD. Among the fresh samples, there were 432.99 copies/µl, 167.92 copies/µl and 249.73 copies/µl in the CD, UC and nIBD groups, respectively. The highest bacterial load was in the CD group.

**CONCLUSION:**

This study does not provide evidence for a role of MAP in the etiology of CD, although MAP DNA was detected in all three patient groups. This is the first report of MAP presence in human intestinal biopsies in Brazil.

## RESUMO

**CONTEXTO E OBJETIVO:**

*Mycobaterium avium* subsp*. paratuberculosis* (MAP) tem atraído o interesse de pesquisadores devido às semelhanças entre a paratuberculose e a doença de Crohn (CD). Este estudo objetivou avaliar a frequência de MAP por meio de cultura, histologia e reação da polimerase em cadeia (PCR), em biópsias intestinais de pacientes brasileiros com CD. PCR quantitativa em tempo real (qRT-PCR) foi realizada nas amostras positivas.

**TIPO DE ESTUDO E LOCAL:**

Estudo transversal analítico com grupo controle realizado em duas universidades federais.

**MÉTODOS:**

Amostras frescas foram coletadas de 25 pacientes; cinco com CD, oito com colite ulcerativa (UC) e 12 controles sem doença inflamatória intestinal (nIBD). Também foram coletadas 149 amostras fixadas em parafina (FFPE): 44 CD, 49 UC e 56 nIBD.

**RESULTADOS:**

Nenhuma das amostras frescas foi positiva para MAP. Cinco amostras FFPE (uma CD, duas UC e duas nIBD) e três amostras frescas (uma de cada grupo) foram positivas por IS*900*-PCR. qRT-PCR foi realizada nessas oito amostras. Nas amostras FFPE, havia 192,12 cópias/µl no grupo CD, 72,28 cópias/µl no UC e 81,43 cópias/µl no nIBD. Nas amostras frescas, havia 432,99 cópias/µl, 167,92 cópias/µl e 249,73 cópias/µl nos grupos CD, UC e nIBD, respectivamente. A maior carga bacteriana foi encontrada no grupo CD.

**CONCLUSÃO:**

Este estudo não fornece evidências do papel de MAP na etiologia da CD, embora DNA de MAP tenha sido detectado em pacientes dos três grupos. Este é o primeiro relato da presença de MAP em biópsias intestinais humanas no Brasil.

## INTRODUCTION

Crohn’s disease (CD) is a chronic inflammatory disease of the human gastrointestinal tract. It has increasing incidence worldwide, and unknown etiology.^1^ Paratuberculosis is a form of chronic granulomatous enteritis, caused by *Mycobacterium avium* subsp. *paratuberculosis* (MAP), which affects all species of ruminants worldwide.^2^ Because of the similarities between paratuberculosis and CD, the possibility of an infectious etiology for this disease has been widely discussed and MAP has attracted the interest of many researchers.^3^^,^^4^

MAP has been detected by means of cultures and the polymerase chain reaction (PCR) in patients with CD.^3^^,^^5^^,^^6^^,^^7^^,^^8^ Numerous theories about a possible cause for CD have been postulated over the years.^9^ Scientific evidence supports the theory of an interaction between a persistent environmental stimulus (such as a microbial antigen) and genetic factors that regulate the immune response and/or function of intestinal mucosa.^10^^,^^11^ However, it would be simplistic to conclude that one agent is solely responsible for the etiology of CD: a multifactorial cause is more likely.^4^^,^^12^

Several studies investigating these causes have been conducted, and they suggest that, although further research is required, an association between MAP and CD cannot be ruled out.^3^^,^^4^^,^^13^ Some researchers believe that most CD cases are not caused by “infection with MAP” although they do not neglect the extremely strong suspicion that MAP plays a role in the pathogenesis of CD.^14^

## OBJECTIVE

The aim of this study was to evaluate the frequency of MAP in fresh and formalin-fixed paraffin-embedded intestinal biopsies, through culture and molecular techniques. The samples were collected from CD patients attending a referral center for treatment of intestinal diseases in the state of Minas Gerais, Brazil.

## METHODS

### Patients and samples

#### Fresh tissues

Fresh samples were collected prospectively and randomly, using sterile biopsy forceps, from patients undergoing routine ileocolonoscopy as part of their normal clinical treatment in the Instituto Alfa de Gastroenterologia (IAG), Hospital das Clínicas (HC), Universidade Federal de Minas Gerais (UFMG), in 2011-2012. Prior to sample collection, informed consent was obtained from each individual. The confirmation of CD in each patient was based on clinical, radiological, endoscopic and histopathological findings. Patients with other inflammatory bowel diseases were included in the ulcerative colitis (UC) group. Patients diagnosed with non-inflammatory bowel disease (nIBD) were those who underwent ileocolonoscopy without a clinicopathological diagnosis of any inflammatory disease. The inclusion criteria for this study were identification of the patient’s condition and informed consent received from the patient.

Samples were collected from 25 patients, comprising five patients with CD, eight with UC and 12 with nIBD. From each patient, six biopsy specimens were collected: three samples from the terminal ileum and three samples from the ascending colon. Four samples from each patient (two ileum and two colon samples) were placed in 1.5 ml microcentrifuge tubes containing Middlebrook OADC broth supplemented with 20% autoclaved glycerol, and were then stored in liquid nitrogen for subsequent microbiological culturing and DNA extraction. The remaining two samples (one ileum and one colon sample) were placed in 1.5 mL microtubes containing buffered formalin for subsequent histological analysis. For patients with CD and UC, samples were collected from inflamed and uninflamed parts of the mucosa.^6^

#### Formalin-fixed paraffin-embedded tissues

We obtained 149 paraffin blocks from biopsies on 143 patients who were attended at HC-UFMG between 2009 and 2011. This material was produced in the Pathology Laboratory of IAG-HC-UFMG. In the same way as with the fresh samples, the paraffin blocks were also categorized into three groups: 56 samples from patients with nIBD, 44 from patients with CD and 49 from patients with UC. From each block, two slides for histopathological analysis were prepared and three slices of 10-20 μm thickness were used for DNA extraction.

Samples were selected by convenience and their power to detect differences was estimated to be between 50% and 80%, according to Pocock’s table^15^ (p1 = 0.04; p2 = 0.10; f(α,β) = 4.17).

#### Tissue processing and MAP cultures

Samples for cultivation were taken to the Bacterial Diseases Laboratory, Universidade Federal de Viçosa (LDBAC-UFV), where they were macerated and decontaminated as described by Bull et al.^6^ and Sechi et al.^8^ Briefly, for decontamination, 0.5 ml of 2% NaOH was added to the samples, and they were left to rest for 20 minutes at room temperature. Subsequently, the samples were centrifuged at 3000 x *g* for 30 minutes; the supernatant was discarded, and the pellet was washed with 10 ml of phosphate-buffered saline (PBS). After washing, the pellet was resuspended in 0.5 ml of TEN buffer (50 μM of Tris-HCl, 100 mM of EDTA and 150 mM of NaCl; pH 8). Aliquots of 100 μl of the suspension were inoculated into four tubes of Herrold Egg Yolk Medium (HEYM): two with mycobactin *J* and two without mycobactin *J*. The remaining 100 μl were inoculated into a tube containing Middlebrook 7H9 broth, supplemented with Middlebrook OADC and mycobactin *J*, and all the tubes were incubated at 37 °C for up to 30 weeks.

### DNA extraction and PCR

For DNA extraction, the Wizard genomic DNA purification kit was used in accordance with the manufacturer’s recommendations and the extracted DNA was stored at 8 °C for later use. PCR reactions were performed on all samples. Go Taq Green Master Mix was used in accordance with the manufacturer’s instructions and the primers BN1 (5’-GTT ATT AAC GAC GCC CAG C-3’) and BN2 (5’-ACG ATG CTG TGT TGG GCG TTA G-3’),^16^ based on the insertion sequence IS*900*, which amplifies a fragment of 626 bp, were used. Each reaction had a total volume of 25 μl, comprising: 12.5 μl of mix, 1 μl of each primer at the initial concentration of 10 pmol/μl, 6.5 μl of ultrapure water and 4 μl of DNA extracted at a concentration of approximately 200 μg/μl. PCR was carried out as recommended by Sivakumar et al.,^16^ i.e. initial denaturation at 94 °C for four minutes, 30 cycles of 94 °C for one minute, 60 °C for one minute, 72 °C for one minute and a final extension step at 72 °C for four minutes.

To confirm the DNA extraction process, PCR reactions were performed to target a region of the human *APC* gene, using the primers F (5’-CCC CTC CAA ATG AGT TAG CTG C-3’) and R (5’-CTCTGC TTT ATT GTC ATC CAA TTC A-3’).^17^ Amplified fragments were viewed by means of electrophoresis, on 1% agarose gel in tris-borate-EDTA (TBE) stained with GelRed nucleic acid gel stain, using an ultraviolet transilluminator. A 100 bp ladder was used as a molecular size marker and ultrapure water was used as a negative control.

### Sequencing and genetic analysis

Amplified fragments were extracted and purified from agarose gel using the Wizard SV gel and PCR clean-up system, in accordance with the manufacturer’s instructions. Subsequently, both strands were sequenced in triplicate. The sequences were edited using the DNAMAN software, and then compared with the sequence of the MAP K-10 strain, which has been deposited in GenBank, using the Basic Local Alignment Search Tool (BLAST) software, which is available from the National Center for Biotechnology Information (NCBI; http://www.ncbi.nlm.nih.gov).

### Histopathological analysis

The samples stored in buffered formalin were processed in accordance with the routine procedures of the Histopathology Laboratory, Veterinary Department, Universidade Federal de Viçosa (DVT/UFV). Paraffin-embedded material was used in preparing slides, which were then stained with Ziehl-Neelsen (ZN) to ascertain whether acid-fast bacilli were present.

### Quantitative real time PCR (qRT-PCR)

qRT-PCR was performed on the samples that were found to be positive through PCR. Reactions with absolute quantitation were performed in duplicate on plates with 48 wells using the TaqMan Universal Master Mix II (Applied Biosystems, Foster City, CA, USA). Genomic quantitation of each sample was generated by means of the detection software through the Eco real-time PCR system, and this was compared with the standard curve of the bacterial genome (10^6^ to 10^1^ copies) using the values of the quantification cycle (Cq) for each reaction. The reaction used the primers MPF (5’-CCG CTA ATT GAG AGA TGC GAT T-3’) and MPR (5’-CCA GAC AGG TTG TGC CAC AA-3’), which were based on the IS*900* insertion sequence and the specific probe (5’-FAM-ACC TCC GTA ACC GTC ATT GTC CAG ATC A-TAMRA-3’).^18^ The initial concentration of the sample for constructing the standard curve was determined by using the following formula, in accordance with the QuantiFast SYBR green PCR handbook (Qiagen, Valencia, CA, USA):

$$molecules/ml\;=\frac{concentration\;of\;DNA\;(g/ml)}{size\;of\;DNA\;(bp)\;x\;660\;x\;6.022\;x\;10^{-23}}$$

For each reaction (total of 20 μl), we used 10 μl of Mix II, 1 μl of each primer at the initial concentration of 10 pmol/μl, 5.5 μl of nuclease-free water, 2 μl of DNA and 0.5 μl of specific probe at the initial concentration of 10 pmol/μl. Amplifications were performed as described by Herthnek et al.,^18^ and are briefly described here: incubation for two minutes at 50 °C followed by activation of polymerase for 10 minutes at 95 °C; after this pretreatment, the samples were subjected to 45 cycles of 95 °C for 15 seconds and 60 °C for one minute.

### Statistical analysis

All the statistical analyses were performed using the Statistica 7.0 software (StatSoft Inc, 2007). The data were subjected to analysis of variance (ANOVA) and means were compared using the F test. In cases of significant differences Tukey’s test was used at 5% probability (P < 0.05).

### Ethical considerations

This study was approved by the Research Ethics Committee of Universidade Federal de Minas Gerais (UFMG) (ETIC no. 0471.0.203.000-10). All participants provided documented informed consent prior to taking part in this study.

## RESULTS

### Patients

Fresh samples were collected from 14 male and 11 female patients, whose mean age was 46.5 years, (range 23-74 years; Table 1). One hundred and forty-nine formalin-fixed paraffin-embedded samples were collected from 143 patients: 56 males and 87 females whose mean age was 40.5 years (range 2-83 years). Six samples were collected from patients from whom samples were taken at two different times. The samples were divided into three groups: 44 from patients with CD, 49 from patients with UC and 56 from patients with nIBD (Table 2).

**Table 1.**
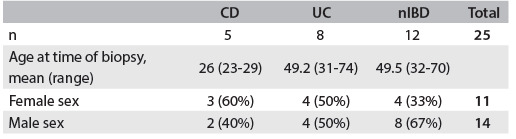
Characteristics of patients with Crohn’s disease (CD), ulcerative colitis (UC) and non-inflammatory bowel disease (nIBD) (controls): fresh samples included in the study

**Table 2.**
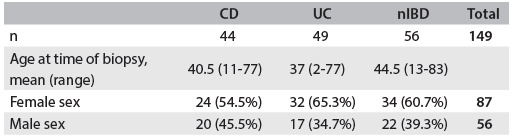
Characteristics of patients with Crohn’s disease (CD), ulcerative colitis (UC) and non-inflammatory bowel disease (nIBD) (controls): formalin-fixed paraffin-embedded samples included in the study

### MAP cultures

Fresh samples from CD, UC or nIBD patients did not provide any positive result for viable MAP with any culture medium used in this study, even after 30 weeks.

### PCR

DNA was extracted successfully from all samples. In five formalin-fixed paraffin-embedded samples, fragments of a size similar to what was expected were amplified by means of PCR: 1/44 (2.3%) from patients with CD, 2/49 (4%) from patients with UC and 2/56 (3.5%) from patients with nIBD. However, these differences between the groups were not statistically significant (Table 3). In three fresh samples, fragments of a size similar to what was expected were amplified: 1/5 (20%) from patients with CD, 1/8 (12.5%) from patients with UC and 1/12 (8.3%) from patients with nIBD. These differences between the groups were not statistically significant (Table 3).

**Table 3.**
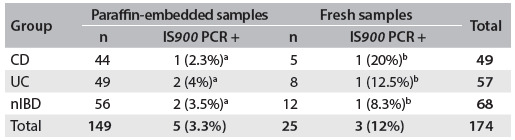
Relationship between IS*900* PCR results and clinical groups of patients

Results followed by the same letters did not differ statistically according to Tukey’s test at 5% probability. Paraffin-embedded samples: F = 0.1211, P = 0.8860; fresh samples: F = 0.2051, P = 0.8161. CD = Crohn’s disease; UC = ulcerative colitis; nIBD = non-inflammatory bowel disease; PCR = polymerase chain reaction.

### Sequencing and genetic analysis

All the amplicons of size 626 bp were sequenced; genetic analysis revealed that these amplicons were 97-99% identical to the sequence of the MAP K-10 strain, which is available in the NCBI database.

### Histopathological analysis

**Figure 1.**
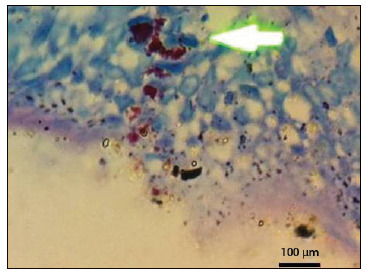
Acid-fast bacilli in the intestinal mucosa of a patient with Crohn’s disease, with Ziehl-Neelsen staining.

Among the formalin-fixed paraffin-embedded samples, acid-fast bacilli were identified (Figure 1) on 15/149 (10%) of the slides stained with ZN. These comprised 9/44 (20.4%) from patients with CD, 1/49 (2%) from patients with UC and 5/56 (8.9%) from patients with nIBD. The mean detection rate for acid-fast bacilli in the CD group was 10 times higher than in the UC group (P < 0.01); there were no statistically significant differences between the CD and nIBD groups, or between the UC and nIBD groups (Table 4). Among the fresh samples, acid-fast bacilli were detected on 1/25 (4%) of the slides, from one patient with UC. Acid-fast bacilli were not identified on slides from patients with CD or nIBD. Among the fresh samples, the differences between the groups were not statistically significant (Table 4).

**Table 4.**
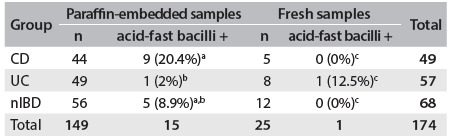
Relationship between presence of acid-fast bacilli and clinical groups of patients included in the study

Results followed by the same letters did not differ statistically according to Tukey’s test at 5% probability. Paraffin-embedded samples: F = 4.5879, P = 0.0117; fresh samples: F = 1.0686, P = 0.3607. CD = Crohn’s disease; UC = ulcerative colitis; nIBD = non-inflammatory bowel disease.

### qRT-PCR

qRT-PCR was performed on eight samples that were positive according to PCR (five formalin-fixed paraffin-embedded and three fresh samples). Among the formalin-fixed paraffin-embedded samples, we observed values of 192.12 copies/μl in the CD group, 72.28 copies/μl in the UC group and 81.43 copies/μl in the nIBD group. Among the fresh samples, we observed values of 432.99 copies/μl, 167.92 copies/μl and 249.73 copies/μl in the CD, UC and nIBD groups, respectively (Table 5).

**Table 5.**
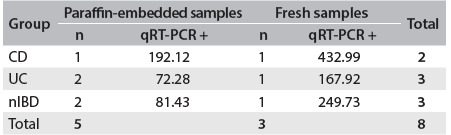
Relationship between qRT-PCR results and clinical groups of patients

CD = Crohn’s disease; UC = ulcerative colitis; nIBD = non-inflammatory bowel disease; PCR = polymerase chain reaction.

## DISCUSSION

Studies that included children have been more likely to report a positive result for MAP than those with an adult population.^3^ Dell’Isola et al.^19^ suggested that if the initial MAP infection occurred during childhood, detection of this infection would be more likely in studies among children. However, in the present study, inclusion of children (formalin-fixed paraffin-embedded samples) did not influence our results.

MAP culturing from human intestinal biopsy material is quite difficult, even under optimal conditions. However, several research groups have been able to grow MAP from tissues from patients with CD, using classical culturing methods, with success rates ranging from 0-40%.^13^

MAP isolates from humans not only present the usual sample decontamination requirements and have very slow growth, but also occur in spheroplasts (a cell wall-deficient form), which are extremely hard to isolate, recover and maintain in sufficient numbers for studies.^20^

Additionally, MAP isolation may also have been negatively affected by the freezing of the samples. It was not possible to work with fresh samples because of the distance between HC/UFMG and LDBAC. Freezing of samples before processing was therefore necessitated and even though a cryoprotectant was used, the ideal would have been for the tissues to have been processed immediately.^6^

Although MAP was not isolated in this study, it is important to highlight that MAP has only been recovered from human tissues with CD. This microorganism has never been isolated from patients with UC or nIBD.^13^

The PCR results regarding MAP detection have been inconclusive and conflicting. Reports have ranged from 0-100% detection in each group (CD, UC and nIBD) using a variety of different methodologies and target sequences.^3^^,^^4^ Contrary to other studies in this field,^5^^,^^6^^,^^7^ MAP was not detected more frequently among our patients with CD than among those with UC or nIBD. However, other research groups have shown that MAP detection in patients with CD occurs more frequently than in patients with UC or nIBD.^21^^,^^22^

In this study, we observed the presence of MAP DNA in intestinal biopsy specimens from eight patients among the 174 samples tested. Previous studies have demonstrated that MAP is difficult to detect reliably and reproducibly by means of PCR on DNA extracted from human tissues.^6^ Thus, the use of non-optimal procedures in processing the samples may result in false-negative results. Bull et al.^6^ indicated several important steps that should be followed in the DNA extraction method, such as processing of fresh tissues (i.e. which have never been frozen); mechanical disturbance to ensure access to MAP DNA; resuspension of the DNA overnight at 4 °C; and nested PCR. In this study, we followed these recommendations wherever possible. However, as previously mentioned, it was necessary for us to freeze the samples, and this could be one reason for the low MAP detection rate in our results.

Few studies have shown higher frequencies of detection of MAP or acid-fast bacilli in patients with CD.^23^^,^^24^^,^^25^^,^^26^ This may be due to the difficulty in detecting MAP in tissue, given that MAP occurs in spheroplast form, and only small quantities of the microorganism are present in the tissues. For as long as these technical limitations remain unresolved, it will continue to be a challenge to demonstrate the presence of MAP in tissues of patients with CD.^13^

Considering the small number of PCR-positive samples that were tested for qRT-PCR, we could not make any statistical inferences about the quantities of DNA found in the three groups (CD, UC and nIBD), although we observed that CD patients had higher bacterial loads in both formalin-fixed paraffin-embedded and fresh samples.

Some studies have supported the theory that MAP is present in most individuals. However, it is found in greater quantities in people with CD, thus suggesting that MAP is an organism that is ubiquitous in the environment and that it is an opportunistic pathogen and not a primary cause of CD.^13^

In this study, the frequency of MAP detection by means of PCR did not differ between CD, UC and nIBD patients, and although the bacterial load was higher in patients with CD, it is not known whether higher bacterial loads cause higher inflammation scores or whether higher inflammation scores cause higher bacterial loads. One possible explanation may be that the microorganism finds better conditions for replication in patients with CD than in patients with UC or nIBD. Immunological factors relating to MAP, in susceptible patients, may allow MAP to replicate in larger quantities, thereby increasing the bacterial load in patients with CD. This is corroborated by Lee et al.,^27^ who showed that MAP gave rise to general colonization of the mucosa and suggested that there was simply an increase in mucosal surface colonization (dysbiosis) in CD cases that was unassociated with causality. Dysbiosis and reduced bacterial diversity of the intestinal microbiome in CD are likely to promote MAP growth and detection.^13^

Further investigations into the etiological role of MAP in CD are needed. Analysis on the human intestinal microbiome in healthy and CD patients would establish whether MAP belongs to the normal human microbiota or not. CD remains a debilitating disease that severely affects the quality of life of its sufferers. Further research is required in order to definitively answer the questions regarding the etiological nature of the disease.

## CONCLUSION

MAP was present in all the groups of patients analyzed, although the greatest bacterial loads were observed in the CD group. This study supports the view that MAP is a ubiquitous organism that colonizes the mucosal surfaces of the gut, thereby resulting in increased detection in CD patients. This study does not provide evidence for any role played by MAP in Crohn’s disease; its role remains controversial and inconclusive. This is the first report on the presence of MAP in biopsy specimens from the human gut in Brazil.

## References

[1] Shanahan F (2002). Crohn’s disease. Lancet.

[2] Chiodini RJ, Van Kruiningen HJ, Merkal RS (1984). Ruminant paratuberculosis (Johne’s disease): the current status and future prospects. Cornell Vet.

[3] Abubakar I, Myhill D, Aliyu SH, Hunter PR (2008). Detection of Mycobacterium avium subspecies paratuberculosis from patients with Crohn’s disease using nucleic acid-based techniques: a systematic review and meta-analysis. Inflamm Bowel Dis.

[4] Feller M, Huwiler K, Stephan R (2007). Mycobacterium avium subspecies paratuberculosis and Crohn’s disease: a systematic review and meta-analysis. Lancet Infect Dis.

[5] Autschbach F, Eisold S, Hinz U (2005). High prevalence of Mycobacterium avium subspecies paratuberculosis IS900 DNA in gut tissues from individuals with Crohn’s disease. Gut.

[6] Bull TJ, McMinn EJ, Sidi-Boumedine K (2003). Detection and verification of Mycobacterium avium subsp. paratuberculosis in fresh ileocolonic mucosal biopsy specimens from individuals with and without Crohn’s disease. J Clin Microbiol.

[7] Naser SA, Ghobrial G, Romero C, Valentine JF (2004). Culture of Mycobacterium avium subspecies paratuberculosis from the blood of patients with Crohn’s disease. Lancet.

[8] Sechi LA, Scanu AM, Molicotti P (2005). Detection and Isolation of Mycobacterium avium subspecies paratuberculosis from intestinal mucosal biopsies of patients with and without Crohn’s disease in Sardinia. Am J Gastroenterol.

[9] Prantera C (2007). Mycobacteria and Crohn’s disease: the endless story. Dig Liver Dis.

[10] Chamberlin W, Graham DY, Hulten K (2001). Review article: Mycobacterium avium subsp. paratuberculosis as one cause of Crohn’s disease. Aliment Pharmacol Ther.

[11] Chiodini RJ, Hermon-Taylor J (1993). The thermal resistance of Mycobacterium paratuberculosis in raw milk under conditions simulating pasteurization. J Vet Diagn Invest.

[12] Sibartie S, Scully P, Keohane J (2010). Mycobacterium avium subsp. Paratuberculosis (MAP) as a modifying factor in Crohn’s disease. Inflamm Bowel Dis.

[13] Chiodini RJ, Chamberlin WM, Sarosiek J, McCallum RW (2012). Crohn’s disease and the mycobacterioses: a quarter century later. Causation or simple association?. Crit Rev Microbiol.

[14] Momotani E, Romona NM, Yoshihara K (2012). Molecular pathogenesis of bovine paratuberculosis and human inflammatory bowel diseases. Vet Immunol Immunopathol.

[15] Pocock SJ (1988). Clinical trials. A practical approach.

[16] Sivakumar P, Tripathi BN, Singh N (2005). Detection of Mycobacterium avium subsp. paratuberculosis in intestinal and lymph node tissues of water buffaloes (Bubalus bubalis) by PCR and bacterial culture. Vet Microbiol.

[17] Rivero ER, Neves AC, Silva-Valenzuela MG, Sousa SO, Nunes FD (2006). Simple salting-out method for DNA extraction from formalin-fixed, paraffin-embedded tissues. Pathol Res Pract.

[18] Herthnek D, Englund S, Willemsen PT, Bölske G (2006). Sensitive detection of Mycobacterium avium subsp. paratuberculosis in bovine semen by real-time PCR. J Appl Microbiol.

[19] Dell’Isola B, Poyart C, Goulet O (1994). Detection of Mycobacterium paratuberculosis by polymerase chain reaction in children with Crohn’s disease. J Infect Dis.

[20] Hines ME, Styer EL (2003). Preliminary characterization of chemically generated Mycobacterium avium subsp. paratuberculosis cell wall deficient forms (spheroplasts). Vet Microbiol.

[21] Rath T, Roderfeld M, Blöcher S (2011). Presence of intestinal Mycobacterium avium subspecies paratuberculosis (MAP) DNA is not associated with altered MMP expression in ulcerative colitis. BMC Gastroenterol.

[22] Ricanek P, Lothe SM, Szpinda I (2010). Paucity of mycobacteria in mucosal bowel biopsies from adults and children with early inflammatory bowel disease. J Crohns Colitis.

[23] Ellingson JL, Cheville JC, Brees D, Miller JM, Cheville NF (2003). Absence of Mycobacterium avium subspecies paratuberculosis components from Crohn’s disease intestinal biopsy tissues. Clin Med Res.

[24] Jeyanathan M, Boutros-Tadros O, Radhi J (2007). Visualization of Mycobacterium avium in Crohn’s tissue by oil-immersion microscopy. Microbes Infect.

[25] Romero C, Hamdi A, Valentine JF, Naser SA (2005). Evaluation of surgical tissue from patients with Crohn’s disease for the presence of Mycobacterium avium subspecies paratuberculosis DNA by in situ hybridization and nested polymerase chain reaction. Inflamm Bowel Dis.

[26] Sechi LA, Mura M, Tanda F (2001). Identification of Mycobacterium avium subsp. paratuberculosis in biopsy specimens from patients with Crohn’s disease identified by in situ hybridization. J Clin Microbiol.

[27] Lee A, Griffiths TA, Parab RS (2011). Association of Mycobacterium avium subspecies paratuberculosis with Crohn Disease in pediatric patients. J Pediatr Gastroenterol Nutr.

